# Optimising Pain Relief in Acute Pancreatitis: An Evidence-Based Approach

**DOI:** 10.3390/jcm15010113

**Published:** 2025-12-24

**Authors:** Cecilie Siggaard Knoph, Sanjay Pandanaboyana

**Affiliations:** 1 Centre for Pancreatic Diseases, Department of Gastroenterology, Aalborg University Hospital, 9000 Aalborg, Denmark; c.siggaard@rn.dk; 2 Department of Internal Medicine, Randers Regional Hospital, 8930 Randers, Denmark; 3 HPB and Transplant Unit, Freeman Hospital, Newcastle upon Tyne NE7 7DN, UK; 4 Population Health Sciences Institute, Newcastle University, Newcastle upon Tyne NE1 7RU, UK

**Keywords:** gastroenterology, hepatobiliary surgery, pancreas, pancreatitis, pain

## Abstract

Pain is a dominant symptom in acute pancreatitis, yet high-level evidence guiding optimal analgesic management in acute pancreatitis has been limited. Emerging evidence suggests a role of non-steroidal anti-inflammatory drugs, including cyclooxygenase-2 inhibitors, and opioids in the management of pain in acute pancreatitis patients. Based on a narrative review of the current literature, we provide an overview of available evidence, give an update on recent studies, and propose a treatment algorithm for pain management in acute pancreatitis, considering pharmacological and non-pharmacological modalities, patient comorbidities, and disease severity. Existing studies are generally limited by small sample sizes, heterogeneity in outcomes, unidimensional pain assessments, and a lack of understanding for the pathophysiology of pain in acute pancreatitis. Future trials should focus on multicentre collaboration, comprehensive pain evaluation, adequate sample sizes, and understanding the complex molecular mechanisms of acute pancreatitis pain.

## 1. Introduction

Pain is the most prominent symptom of the inflammatory disease acute pancreatitis (AP), often being severe and exacerbated by oral feeding [1]. The severity and duration of pain is associated with the disease severity of AP and greatly contributes to patient distress during admission [2,3]. Despite its clinical significance, the pathophysiology of pain in AP remains incompletely understood.

Traditionally, pancreatic pain has been associated with local inflammation and surrounding tissue damage including micro thrombosis, oedema, increased ductal pressure, abdominal hypertension, and ischemia leading to necrosis [4,5]. The early phase of AP is characterised by a severe systemic inflammatory response characterised by increased vascular permeability leading to intravascular hypovolaemia and organ hypoperfusion, which may promote intrapancreatic necrosis and leads to multi-organ failure [6]. Microcirculatory failure may in turn lead to ischemia, and with reperfusion the damage is potentially accelerated. Ischemia–reperfusion and local damage is associated with mitochondrial dysfunction due to intracellular calcium overload in acinar cells, which promotes premature enzyme activation, cellular necrosis, and amplification of inflammatory signalling [7,8]. These molecular mechanisms highlight the importance of fluid resuscitation in the early phases of AP [9,10].

The necrotic acinar cells within the pancreas release damage-associated molecular patterns including ATP, trypsin, and kallikrein, which activate stellate cells and macrophages. The activation of stellate cells, via bradykinin receptors, exacerbates acinar cell injury, thereby worsening local damage by autodigestion within the gland. This creates a self-continuing cycle of necrotic injury and pain [5]. Extracellular ATP also acts as a danger signal by activating receptors on immune cells and sensory neurons, thereby increasing inflammation and pain signalling [5,11]. Furthermore, the extensive release of inflammatory mediators in AP may contribute to primary hyperalgesia by the activation of sensory neurons [11,12]. The activated sensory neurons release neuropeptides such as substance P and calcitonin gene-related peptide, which enhances pain signals by increasing spinal cord excitability, thus creating an “auto-amplification loop” of pain and inflammation [11]. Understanding these molecular and neurogenic mechanisms provides insight into why pain in AP is often severe and difficult to control, highlighting the importance of effective analgesia.

Pain management has previously been identified as a number one priority in AP by health professionals and patients [13]. From the perioperative setting, uncontrolled pain has been implicated in increased risk of thromboembolic events, immunosuppression, and hypoventilation [14,15,16,17]. These physiological responses to pain have been shown to decrease with pain relief in post-operative settings [15,18,19]. In AP, insufficient pain management may also delay oral refeeding and prevent remobilization, thereby worsening the disease course further. Furthermore, prolonged pain can potentially lead to sensitization within the central nervous system, increasing the risk of the chronification of pain—especially in patients with repeated episodes of AP [20].

Traditionally, guidelines on AP management have neglected to mention pain management, although some guidelines have highlighted the importance of gaining pain control in patients with AP [9,21,22,23]. Identifying the most effective treatment for pain in AP has been challenging, as available studies are generally small, heterogeneous, and methodologically diverse, likely reflecting the difficulty of conducting studies in patients with AP. Furthermore, it has been suggested that the treatment choice itself may influence the disease course, with several studies indicating potential deterioration or amelioration of AP with different analgesic treatment modalities. However, recently there has been an increased focus on pain management strategies in AP with several studies emerging—including well-designed randomised, controlled trials—and even upcoming guidelines supported by the United European Gastroenterology and the European Pancreatic Club [24]. The purpose of this narrative review is to provide an overview of the existing literature on the efficacy and safety of different analgesia for achieving pain relief in AP, give an update on the most recent advances in the field, and propose an evidence-based algorithm for the future management of pain in AP patients.

## 2. Non-Steroidal Anti-Inflammatory Drugs

The WHO ladder has often been recommended for pain management in AP based on the management of pain in the perioperative setting [25]. In this framework, simple analgesics like paracetamol and non-steroidal anti-inflammatory drugs (NSAIDs) are first-line therapy. If pain relief is insufficient, pain therapy may be escalated by first adding weak opioids and thereafter strong opioids [26]. In keeping with these recommendations, an observational study found that simple analgesics were most often the first choice for treating AP pain in Europe and Australia. In Asia, simple analgesics were the first choice in 39% of patients admitted with first-time AP [27].

Non-selective NSAIDs have been compared to non-NSAIDs (most often opioids) in several randomised trials for pain relief, as measured by the need for rescue analgesia and pain intensity, including pain-free intervals, in AP patients. As such, a randomised trial of 30 AP patients found a reduction in pain intensity, number of days with pain, and need for opioid injections with 7 days of treatment with rectal indomethacin compared to placebo [28]. Another three-arm randomised trial of 90 AP patients compared intravenous treatment with the NSAID dexketoprofen, paracetamol, or tramadol and found similar pain relief, as measured by the visual analogous scale (VAS), after 30 min. Furthermore, there was no significant difference in need for rescue morphine [29]. Likewise, there was no significant difference in VAS scores within one hour, need for rescue analgesia, or days with pain between AP patients (n = 46) receiving diclofenac versus tramadol [30]. On the other hand, in a recent randomised trial of 48 AP patients, intravenous buprenorphine was superior for reducing the need for rescue fentanyl, prolonging pain-free intervals and reducing VAS scores after 24, 48, and 72 h compared to intravenous diclofenac [31]. Interestingly, these findings were persistent upon subgroup analysis in patients with moderately severe or severe AP. Another randomised trial found that intravenous pentazocine reduced the need for rescue fentanyl and prolonged pain-free intervals compared to intravenous diclofenac [32]. Taken together, a recent meta-analysis pooling the evidence of these randomised trials found no difference in pain relief assessed by VAS scores or need for rescue analgesia between non-selective NSAIDs and opioids [33].

The clinical safety profile of NSAIDs with increased risk of gastrointestinal bleeding, cardiac events, and renal injury has been an ongoing concern with NSAID treatment in AP [34]. The above-mentioned studies [28,29,30,31,32] and two other randomised trials [35,36] also assessed the effect of non-selective NSAID treatment on clinical outcomes in AP patients. As such, rectal indomethacin did not increase the risk of gastrointestinal bleeding compared to placebo [28,35]. Furthermore, there was no statistically increased risk of adverse effects of non-selective NSAIDs (dexketoprofen, diclofenac) compared to opioids (tramadol, pentazocine, buprenorphine) [29,30,31,32]. The most common adverse effects reported in these studies were constipation, nausea, vomiting, dyspepsia, somnolence, dizziness, and headache—some of which are more likely related to the safety profile of the comparative opioid. In four of the studies, no difference was observed in the incidence of pancreatic necrosis between AP patients receiving NSAIDs and opioids [31,32,35,36]. Although not powered for such comparison, three studies reported no increase in the occurrence of moderately severe or severe AP with NSAIDs [31,35,36]. Consistently, on meta-analysis, there was no difference in the occurrence of severe AP, pancreatic necrosis, admission time, or mortality with non-selective NSAIDs compared to non-NSAIDs [33]. In this regard, it is worth noting that the evidence on non-selective NSAIDs in AP is based on studies with restricted sample sizes comparing small groups (n = 14–30), heterogenous methodology, and different outcomes. Furthermore, the analgesic potency of NSAIDs is lower than opioids and may therefore result in an increased need for a rescue analgesic in the NSAID groups.

Interestingly, the evidence for cyclooxygenase-2 (COX-2) inhibitors has pointed in another direction and indicates a protective role against progression towards severe AP, as reported in the same meta-analysis [33]. This meta-analysis was based on only one randomised trial comparing sequential treatment with parecoxib and celecoxib with standard of care [37]. In this study, Huang et al. showed a reduction in the occurrence of severe AP, lower levels of inflammatory markers, and a decreased need for rescue analgesia with the COX-2 inhibitor regimen compared to standard of care in AP patients with predicted severe disease (based on an Acute Physiology and Chronic Health Evaluation II (APACHE II) score ≥ 8) [37]. These findings have recently been confirmed in a randomised, controlled trial of 348 patients with predicted severe AP (APACHE II score ≥ 7 or modified Marshall Score ≥ 2) receiving parecoxib with imrecoxib or placebo [38]. In this study, the COX-2 inhibitor regimen reduced severe AP and the duration of organ failure. There was no difference in renal failure or need for dialysis, despite concerns that COX-2 inhibition could impair renal perfusion [39]. Patients with baseline creatinine ≥ 2 times upper limit were excluded, but it was unclear how the deterioration of renal function during study participation was handled. In conclusion, COX-2 inhibitors have shown promising potential as an early disease modifying agent in AP. However, it should be noted that parecoxib treatment does not have approval from major regulatory authorities [40].

Furthermore, it is important to note that, during an attack of AP, renal perfusion becomes prostaglandin-dependent due to third-space fluid depletion, intravascular hypovolaemia, and systemic vasodilation. Inhibiting the COX-2 pathway reduces prostaglandin synthesis and thereby predisposes AP patients to acute renal injury. As such, treatment with any NSAID during AP should be reserved for patients with near-normal renal function and renal function should be monitored closely during treatment.

## 3. Opioids

Several studies have found that opioids are often utilised for pain in AP, likely reflecting the severe intensity of AP pain [27,41,42]. As discussed above, several randomised trials have compared the efficacy of non-selective NSAIDs versus opioids. Two studies reported similar VAS scores after 30–60 min between diclofenac or dexketoprofen versus tramadol [29,30], in contrast with two other studies showing prolonged pain-free intervals and a reduced need for rescue analgesia with opioids buprenorphine or pentazocine compared to diclofenac [31,32]. In addition to these studies, one study of 40 AP patients found a lower need for rescue analgesia and reduced VAS scores with buprenorphine compared to procaine within a three-day study period [43]. Likewise, another study found similar results for pentazocine compared to procaine in AP patients [44]. On the other hand, a small study of just 16 AP patients found that more patients achieved pain relief from metamizole treatment compared to morphine [45]. Some studies have also compared the opioid pethidine with other opioids (hydromorphone, transdermal fentanyl, or buprenorphine) and found similar pain relief as measured by the intensity and duration of pain before and up to 72 h after analgesia administration [46,47,48]. Altogether, systematic reviews and meta-analyses pooling the evidence from these studies have noted a decreased need for rescue analgesia with opioid-based therapies [49,50,51,52], and commented that opioids are almost exclusively used as rescue analgesia in AP trials [53]. Direct comparisons of opioid-based therapies with non-opioid therapies are inherently challenging due to the risk of insufficient pain management in the non-opioid group, and poses a great challenge with pain management studies in AP. Despite the reduction in rescue analgesia noted above, the reviews maintained that current evidence indicates similar pain relief with opioid versus non-opioid regimens [49,50,51,52,53,54]. However, there is a lack of high-quality evidence and current studies are generally underpowered, heterogeneous, and report widely differing outcomes. The opioids included in these studies were pentazocine, buprenorphine, morphine, tramadol, hydromorphone, fentanyl, and pethidine. These opioids have very different opioid-receptor affinities, underlining a fundamental problem with comparing these studies directly. As such, pentazocine, with primary κ-opioid receptor affinity, confers distinct therapeutic effects from buprenorphine with broader activity across µ-, δ-, and κ-receptors [55].

The safety of opioids has been immensely debated in AP due to the risk of disease deterioration from adverse effects like respiratory depression, hypotension, bradycardia, and sedation, which may be particularly detrimental to patients with severe AP who are at risk of organ failure. Furthermore, opioids have a number of effects on the gastrointestinal tract, including dysmotility, sphincter of Oddi spams, and impaired barrier function [56,57,58,59]. These concerns have been reinforced by preclinical studies indicating worsening of AP with opioids. As such, a preclinical study of experimental AP induced in mice by caerulein, L-arginine, or ethanol–palmitoleic found increased necrosis with concomitant morphine injection. This was reversed with naltrexone and in µ-opioid receptor knockout mice [60]. On the other hand, a study of experimental AP in rats found that the effect of morphine on AP severity was insignificant, whereas fentanyl appeared beneficial when administered post-AP induction [61]. The results of these preclinical studies should be taken with caution, since it remains unknown whether the conditions of experimental AP in rats or mice are translational to clinical AP conditions—especially considering that there are interspecies differences in the distribution and function of opioids in the gastrointestinal tract [62]. Retrospective studies have reported adverse outcomes, such as increased admission length, dysmotility, and aggravated morphological disease severity assessed using computed tomography scans, in AP patients on opioid therapy compared to non-opioid therapy [41,63,64]. Another retrospective single centre study found that opioids increased the risk of a composite endpoint of 30-day mortality, the need for invasive ventilation, abdominal surgery, or vasopressor treatment [65]. Consistently, our recent prospective observational studies from the PAINAP database have also found associations between moderately severe to severe AP and opioid therapy—but only with late administration, longer duration, or higher opioid doses [2,66]. Furthermore, we showed—in the same cohort—that more severe pain was associated with more severe AP [3]. Taken together, this suggests reverse causation bias, in which pain severity may act as an underlying confounder for the relationship between opioid therapy and severe AP.

Nevertheless, these observations underscore the importance of careful opioid stewardship in the management of pain in AP. In routine clinical care, pain severity should guide analgesic selection and dosing. Additionally, agent-specific properties—such as receptor affinity, the potential for organ-specific adverse effects, and the risk of gastrointestinal complications—should be considered when choosing an opioid. For example, tramadol, with weaker µ-opioid activity, may be preferred in patients at risk of biliary spasm. Opioid therapy may necessitate dose escalation over time and has been associated with opioid-induced hyperalgesia—a paradoxical increase in pain sensitivity—which may complicate pain assessment and prolong opioid exposure. Furthermore, given the gastrointestinal effects of opioids, analgesic strategies in AP should ideally include the clinical monitoring of gastrointestinal function and a predefined plan for dose reduction or tapering as pain improves.

Based on the assumption that the peripheral effects of opioids may worsen AP disease severity, we conducted a randomised trial in 105 AP patients with predicted severe disease (based on the systemic inflammatory response syndrome), comparing 5 days of methylnaltrexone (a peripheral opioid antagonist) treatment with placebo. In this study, we found no difference in disease severity (measured by the Pancreatitis Activity Scoring System) or gastrointestinal transit time, indicating that, within the complex pathophysiology of AP, where multiple factors concurrently influence gastrointestinal function and disease progression, the contribution of short-term opioid use is insignificant [67,68]. Less is known about the potential risk associated with chronic opioid use due to dependency. Several studies have reported that a substantial number of patients admitted with first-time AP are discharged with an opioid-prescription, especially in cases of pancreatic necrosis [27,69,70]. Although this issue appears most pronounced in recurrent or chronic pancreatitis, it remains an important consideration for managing pain with opioids in patients with AP [71].

## 4. Other Modalities

Observational studies have advocated that epidural analgesia may be a safe alternative for pain management in patients with severe AP due to the decreased risk of respiratory depression and renal injury [72]. This is especially relevant in patients with severe AP and multi-organ failure, where other analgesic modalities are often ineffective or contraindicated. A retrospective cohort study found lower 30-day mortality in critically ill AP patients treated with thoracic epidural analgesia compared to standard of care [73]. Likewise, another retrospective study found a lower incidence of respiratory distress syndrome, the need for mechanical ventilation, renal injury, the need for dialysis, and mortality with thoracic epidural analgesia in patients with severe AP [74]. Still, AP patients rarely receive epidural analgesia during admission [27,75]. A randomised trial found improved pain relief and pancreatic perfusion, measured using computed tomography, with epidural analgesia compared to patient-controlled analgesia in AP patients [76]. Theoretically, this may protect against pancreatic necrosis. However, the same study did not find any difference in the need for necrosectomy. Based on a non-significant decrease in the need for intubation [76], the EPIPAN study hypothesised that epidural analgesia may increase the number of ventilator-free days in AP patients compared to standard of care, but found no difference between groups [77]. Likewise, another randomised trial did not find any clinical benefit, including length of admission, need for mechanical ventilation, or mortality, of epidural block in AP patients [78]. Other nerve blocks, including erector spinae or transversus abdominis plane block and paravertebral nerve blockade, have been reported successful in AP, but these have not been systematically assessed in clinical trials [79,80,81,82].

Another method for administering pain relief is patient-controlled analgesia, allowing patients to escalate analgesia according to the subjective level of pain, which is common in peri- and post-operative pain. In a retrospective cohort study, patient-controlled analgesia was associated with poorer outcomes compared to standard care [83]. Likewise, a randomised trial comparing physician-directed pethidine with patient-controlled hydromorphone found no significant difference in VAS scores and higher rates of severe disease, acute peripancreatic fluid collections, and an increasing need for opioid therapy [48]. As such, patient-controlled analgesia is currently not recommended specifically for AP patients.

Adjuvant analgesics, such as anticonvulsants (e.g., pregabalin) and anxiolytics (e.g., diazepam), have been recommended for persistent pain in chronic pancreatitis [84,85]. However, the evidence for the use of such modalities in AP is limited and confined to pre-clinical settings. In rats, it has been shown that intrathecal gabapentin and low-dose morphine significantly reduced pain-related behaviours, whereas gabapentin or low-dose morphine by itself did not [86]. Similarly, another study into experimental AP in rats has suggested that the coadministration of a low-dose NMDA receptor antagonist may potentiate the analgesic effect of morphine [87]. Furthermore, the intraperitoneal or intrathecal administration of bradykinin B2 receptor antagonists have been shown to reduce pain in experimental AP [88]. The underlying mechanism may involve reduced stellate cell activation, which interrupts the feedback loop between acinar cell injury and immune activation that drives pain and inflammation in the early phases of AP, as described above. Finally, diazepam ameliorated oedema in experimental AP when administered pre-induction of AP [89]. Some studies have also examined the effects of Chinese herbal medicine and acupuncture on pain and gastrointestinal symptoms in AP, indicating better pain relief and reduced time to oral feeding with traditional and electroacupuncture, as well as Chinese herbal medicine formulas Bupleurum and Scutellaria Purgative Decoction [90,91,92].

## 5. Treatment Algorithm

**Figure 1:**
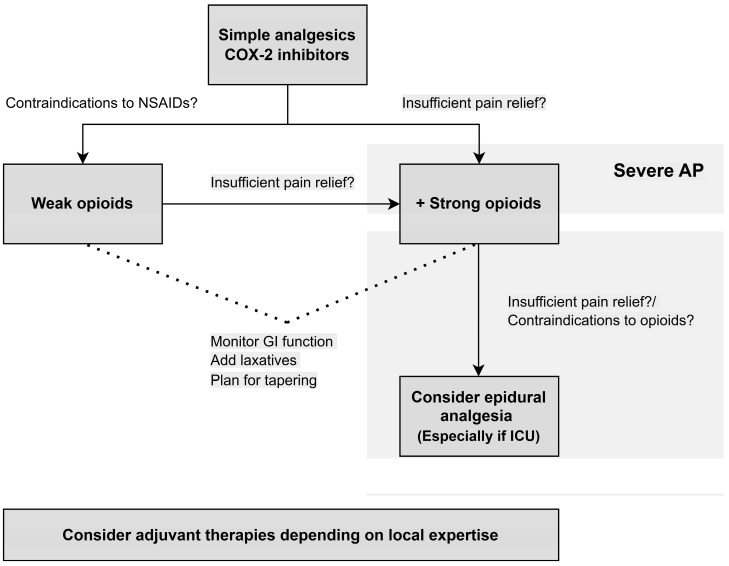
Treatment algorithm for pain management in acute pancreatitis. COX-2, cyclooxygenase-2; NSAIDs, non-steroidal anti-inflammatory drugs; GI, gastrointestinal; ICU, intensive care unit.

According to the evidence described above, we present an algorithm for the treatment of pain in AP (Figure 1). The algorithm is based on the following statements:

1. COX-2 inhibitors decrease the risk of severe AP in patients with no contraindications to NSAIDs.
2. Opioid-based therapies decrease the need for rescue analgesia.
3. Opioids are safe for AP patients. Due to the risk of dependency, physicians should have a plan for tapering.
4. Epidural analgesia is a safe alternative and may improve pain relief for patients with severe AP requiring admission to the intensive care unit or for patients with contraindications to opioid treatment.
5. Adjuvant therapies, such as nerve blocks and acupuncture, should be considered in all patients depending on local expertise for add-on effect.
6. In patients with severe AP, including organ failure, pain control should be prioritised and strong opioids, alternatively epidural analgesia, should be started upfront.

## 6. Future Directions

There is a lack of high-level evidence in the field of pain management for patients with AP. As described above, the studies available are limited by low sample sizes, diverse methodology, and high variability in outcomes reported. Thus, there is still a need for well-designed randomised trials looking into the efficacy and safety of different analgesic modalities, as well as different treatment algorithms. To improve the quality and comparability of future research, study outcomes need to be streamlined and consistently reported.

The unidimensional assessment of pain intensity is often used for the evaluation of pain in AP without reflecting the multifaceted nature of pain in these patients, and, for future research, more comprehensive assessment tools should be considered [93,94]. Severe abdominal pain is associated with severe AP, but does not predict severity very well [3]. Still, insufficiently treated pain can contribute to immobilisation, delayed enteral feeding, and neurohumoral stress responses, potentially predisposing patients to poorer outcomes [14,15,16,17,95]. Existing studies have not selected patients based on pain severity, meaning that the inclusion of patients with mild pain may have diluted the potential beneficial effects on pain management of the analgesic agents studied. Future studies may consider evaluating differentiated algorithms for the management of mild versus severe pain in AP. Furthermore, some studies have investigated the potential disease-modifying effects of different analgesic modalities, including NSAIDs and opioids, as discussed above. This is particularly challenging in AP, where a high proportion of patients have mild, self-limiting disease and a smaller proportion of patients rapidly develop life-treating disease with multi-organ failure. Furthermore, the scoring systems available for predicting severe AP have performed poorly [96]. This heterogeneity in disease course across patients and the challenges with prediction must be considered in sample size calculations to avoid underpowered studies. The optimal settings for such studies are multicentre collaboration studies to secure sufficiently large and representative cohorts.

## 7. Conclusions

In conclusion, effective pain management is a cornerstone in the treatment of acute pancreatitis. Recent evidence suggests that COX-2 inhibitors may prevent progression towards severe AP while providing pain relief. In meta-analyses, opioids reduced the need for rescue analgesia and the current evidence suggests that short-term opioid treatment is safe in the setting of AP. Other analgesic modalities are available and safe in AP, including epidural analgesia, nerve blocks, acupuncture, and Chinese herbal medicine. Based on the current evidence, we suggested an evidence-based algorithm for the future management of pain in AP. Importantly, future studies’ strategies should not only be evaluated in larger, high-quality, multicentre trials but should also focus on treatments that are rationally aligned with the current understanding of AP pathophysiology, targeting mechanisms that drive pain, inflammation, and disease progression. The current evidence available is limited by small sample sizes and diverse methodology, highlighting the need for high-quality, multicentre, randomised trials to refine strategies. Importantly, future studies should aim to improve the understanding of the pathophysiology of acute pancreatic pain so that treatment strategies can be rationally aligned with the underlying mechanisms that drive pain, inflammation, and disease progression in AP.

## Data Availability

No new data were created or analysed in this study. Data sharing is not applicable to this article.

## Abbreviations

The following abbreviations are used in this manuscript:

AP	Acute Pancreatitis
NSAIDs	Non-Steroidal Anti-Inflammatory Drugs
VAS	Visual Analogous Scale
COX-2	Cyclooxygenase-2
APACHE-II	Acute Physiology and Chronic Health Evaluation II

## References

[1] Banks P.A., Bollen T.L., Dervenis C., Gooszen H.G., Johnson C.D., Sarr M.G., Tsiotos G.G., Vege S.S., Acute Pancreatitis Classification Working Group (2013). Classification of acute pancreatitis—2012: Revision of the Atlanta classification and definitions by international consensus. Gut.

[2] Pandanaboyana S., Siggaard Knoph C., Kuhlmann L., Forget P., Pogatzki-Zahn E., Huang W., Bonovas S., Piovani D., Scheers I., Cardenas Jean K. (2024). Opioid analgesia and severity of acute pancreatitis: An international multicentre cohort study on pain management in acute pancreatitis. United Eur. Gastroenterol. J..

[3] Knoph C.S., Pandanaboyana S., Drewes A.M., Kuhlmann L., Joseph N., Windsor J., Olsen S.S., Varghese C., Huang W., Dhar J. (2025). Pain intensity and prognosis of acute pancreatitis in an international, prospective study. Br. J. Surg..

[4] Fasanell K.E., Davis B., Lyons J., Chen Z., Lee K.K., Slivka A., Whitcomb D.C. (2007). Pain in Chronic Pancreatitis and Pancreatic Cancer. Gastroenterol. Clin. N. Am..

[5] Petersen O.H., Gerasimenko J.V., Gerasimenko O.V., Gryshchenko O., Peng S. (2021). The roles of calcium and ATP in the physiology and pathology of the exocrine pancreas. Physiol. Rev..

[6] Szatmary P. (2022). Acute Pancreatitis: Diagnosis and Treatment. Drugs.

[7] Criddle D.N. (2016). Reactive oxygen species, Ca^2+^ stores and acute pancreatitis; a step closer to therapy?. Cell Calcium.

[8] Menger M.D., Bonkhoff H., Vollmar B. (1996). Ischemia-reperfusion-induced pancreatic microvascular injury. An intravital fluorescence microscopic study in rats. Dig. Dis. Sci..

[9] (2013). Working Group IAP/APA Acute Pancreatitis Guidelines. IAP/APA evidence-based guidelines for the management of acute pancreatitis. Pancreatology.

[10] De-Madaria E., Buxbaum J.L., Maisonneuve P., García García de Paredes A., Zapater P., Guilabert L., Vaillo-Rocamora A., Rodríguez-Gandía M.Á., Donate-Ortega J., Lozada-Hernández E.E. (2022). Aggressive or Moderate Fluid Resuscitation in Acute Pancreatitis. N. Engl. J. Med..

[11] Wu Y., Han C., Luo R., Cai W., Xia Q., Jiang R., Ferdek P.E., Liu T., Huang W. (2023). Molecular mechanisms of pain in acute pancreatitis: Recent basic research advances and therapeutic implications. Front. Mol. Neurosci..

[12] Ceppa E.P., Lyo V., Grady E.F., Knecht W., Grahn S., Peterson A., Bunnett N.W., Kirkwood K.S., Cattaruzza F. (2011). Serine proteases mediate inflammatory pain in acute pancreatitis. Am. J. Physiol. Gastrointest. Liver Physiol..

[13] Mitra V., Munnelly S., Grammatikopoulos T., Mole D., Hopper A., Ryan B., Phillips M., Tarpey M., Leeds J. (2023). The top 10 research priorities for pancreatitis: Findings from a James Lind Alliance priority setting partnership. Lancet Gastroenterol. Hepatol..

[14] Marik P.E., Raghavan M. (2004). Stress-hyperglycemia, insulin and immunomodulation in sepsis. Intensive Care Med..

[15] Ljungqvist O., Nygren J., Soop M., Thorell A. (2005). Metabolic perioperative management: Novel concepts. Curr. Opin. Crit. Care.

[16] Rosenfeld B.A., Faraday N., Campbell D., Dise K., Bell W., Goldschmidt P. (1994). Hemostatic effects of stress hormone infusion. Anesthesiology.

[17] Vassilakopoulos T., Mastora Z., Katsaounou P., Doukas G., Klimopoulos S., Roussos C., Zakynthinos S. (2000). Contribution of pain to inspiratory muscle dysfunction after upper abdominal surgery: A randomized controlled trial. Am. J. Respir. Crit. Care Med..

[18] Beaussier M., Genty T., Lescot T., Aissou M. (2014). Influence of pain on postoperative ventilatory disturbances. Management and expected benefits. Ann. Franç. Anesth. Réanim..

[19] Yardeni I.Z., Shavit Y., Bessler H., Mayburd E., Grinevich G., Beilin B. (2007). Comparison of postoperative pain management techniques on endocrine response to surgery: A randomised controlled trial. Int. J. Surg..

[20] Arendt-Nielsen L., Morlion B., Perrot S., Dahan A., Dickenson A., Kress H.G.G., Wells C., Bouhassira D., Mohr Drewes A. (2018). Assessment and manifestation of central sensitisation across different chronic pain conditions. Eur. J. Pain.

[21] Tenner S., Vege S.S., Sheth S.G., Sauer B., Yang A., Conwell D.L., Yadlapati R.H., Gardner T.B. (2024). American College of Gastroenterology Guidelines: Management of Acute Pancreatitis. Am. J. Gastroenterol..

[22] Leppäniemi A., Tolonen M., Tarasconi A., Segovia-Lohse H., Gamberini E., Kirkpatrick A.W., Ball C.G., Parry N., Sartelli M., Wolbrink D. (2019). 2019 WSES guidelines for the management of severe acute pancreatitis. World J. Emerg. Surg..

[23] Yokoe M., Takada T., Mayumi T., Yoshida M., Isaji S., Wada K., Itoi T., Sata N., Gabata T., Igarashi H. (2015). Japanese guidelines for the management of acute pancreatitis: Japanese Guidelines 2015. J. Hepato-Biliary-Pancreat. Sci..

[24] Pandanaboyana S., Siggaard Knoph C., Kuhlmann L., Forget P., Pogatzki-Zahn E., Huang W., Bonovas S., Piovani D., Scheers I., Cardenas Jean K. (2025). European Interdisciplinary Guidelines on Pain Management in Acute Pancreatitis: UEG, EPC, EDS, ESDO, EAGEN, ESPGHAN, ESGAR, and ESPCG evidence-based recommendations Acute Pancreatitis Multidisciplinary Group. United European Gastroenterol. J..

[25] Lankisch P.G., Apte M., Banks P.A. (2015). Acute pancreatitis. Lancet.

[26] Ventafridda V., Saita L., Ripamonti C., De Conno F. (1985). WHO guidelines for the use of analgesics in cancer pain. Int. J. Tissue React..

[27] Knoph C.S., Lucocq J., Kamarajah S.K., Olesen S.S., Jones M., Samanta J., Talukdar R., Capurso G., De-Madaria E., Yadav D. (2024). Global trends in opioid use for pain management in acute pancreatitis: A multicentre prospective observational study. United Eur. Gastroenterol. J..

[28] Ebbehøj N., Friis J., Svendsen L.B., Bülow S., Madsen P. (1985). Indomethacin treatment of acute pancreatitis: A controlled double-blind trial. Scand. J. Gastroenterol..

[29] Gülen B., Dur A., Serinken M., Karcioğlu Ö., Sönmez E. (2016). Pain treatment in patients with acute pancreatitis: A randomized controlled trial. Turk. J. Gastroenterol..

[30] Kumar N.S., Muktesh G., Samra T., Sarma P., Samanta J., Sinha S.K., Dhaka N., Yadav T.D., Gupta V., Kochhar R. (2020). Comparison of efficacy of diclofenac and tramadol in relieving pain in patients of acute pancreatitis: A randomized parallel group double blind active controlled pilot study. Eur. J. Pain.

[31] Saini M., Samanta J., Kumar A., Choudhury A., Dhar J., Jafra A., Chauhan R., Muktesh G., Gupta P., Gupta V. (2023). Buprenorphine Versus Diclofenac for Pain Relief in Acute Pancreatitis: A Double-Blinded Randomized Controlled Trial. Clin. Gastroenterol. Hepatol..

[32] Mahapatra S.J., Jain S., Bopanna S., Gupta S., Singh P., Trikha A., Sreenivas V., Garg P.K. (2019). Pentazocine, a Kappa-Opioid AgonistIs Better Than Diclofenac for Analgesia in Acute Pancreatitis: A Randomized Controlled Trial. Am. J. Gastroenterol..

[33] Vargas A., Dutta P., Hawa F., Quingalahua E., Marin R., Vilela A., Nix T., Mendoza-Ladd A., Wilcox C.M., Chalhoub J.M. (2025). Effect of selective COX-2 inhibitors and non-selective non-steroidal anti-inflammatory drugs on severity of acute pancreatitis: A systematic review and meta-analysis. Pancreatology.

[34] Capurso G., Malesci A. (2024). Targeting inflammation to prevent severe acute pancreatitis: NSAIDs are not the holy grail. Dig. Liver Dis..

[35] Machicado J.D., Mounzer R., Paragomi P., Pothoulakis I., Hart P.A., Conwell D.L., De-Madaria E., Greer P., Yadav D., Whitcomb D.C. (2021). Rectal Indomethacin Does Not Mitigate the Systemic Inflammatory Response Syndrome in Acute Pancreatitis: A Randomized Trial. Clin. Transl. Gastroenterol..

[36] Shariatpanahi Z.V., Shahbazi S., Shahbazi E. (2022). Ketorolac and Predicted Severe Acute Pancreatitis: A Randomized, Controlled Clinical Trial. Clin. Med. Res..

[37] Huang Z., Ma X., Jia X., Wang R., Liu L., Zhang M., Wan X., Tang C., Huang L. (2020). Prevention of Severe Acute Pancreatitis with Cyclooxygenase-2 Inhibitors: A Randomized Controlled Clinical Trial. Am. J. Gastroenterol..

[38] Huang L., Feng Z., Yang W., Zhu Y., Li J., Huang L., Wang R., Peng L., He M., Tang Y. (2025). Parecoxib sequential with imrecoxib for occurrence and remission of severe acute pancreatitis: A multicentre, double-blind, randomised, placebo-controlled trial. Gut.

[39] Mahapatra S.J., Garg P. (2020). Inhibition of Cyclooxygenase-2 Pathway: ‘Ice’ for Burning Pancreas?. Am. J. Gastroenterol..

[40] Lin J., Cai W., Kattakayam A., He W., Ke L., Szatmary P., Liu T., Singh V., Mukherjee R., Huang W. (2025). Pharmacological trials of early intervention in predicted severe acute pancreatitis: Implications for therapeutic window and core outcome set. Gut.

[41] Wu B.U., Butler R.K., Chen W. (2019). Factors Associated with Opioid Use in Patients Hospitalized for Acute Pancreatitis. JAMA Netw. Open.

[42] Matta B., Gougol A., Gao X., Reddy N., Talukdar R., Kochhar R., Goenka M.K., Gulla A., Gonzalez J.A., Singh V.K. (2020). Worldwide Variations in Demographics, Management, and Outcomes of Acute Pancreatitis. Clin. Gastroenterol. Hepatol..

[43] Jakobs R., Adamek M.U., von Bubnoff A.C., Riemann J.F. (2000). Buprenorphine or procaine for pain relief in acute pancreatitis. A prospective randomized study. Scand. J. Gastroenterol..

[44] Kahl S., Zimmermann S., Pross M., Schulz H.U., Schmidt U., Malfertheiner P. (2004). Procaine hydrochloride fails to relieve pain in patients with acute pancreatitis. Digestion.

[45] Peiró A.M., Martínez J., Martinez E., de Madaria E., Llorens P., Horga J.F., Pérez-Mateo M. (2008). Efficacy and Tolerance of Metamizole versus Morphine for Acute Pancreatitis Pain. Pancreatology.

[46] Blamey S.L., Finlay I.G., Carter D.C., Imrie C.W. (1984). Analgesia in acute pancreatitis: Comparison of buprenorphine and pethidine. Br. Med. J. Clin. Res. Ed..

[47] Stevens M., Esler R., Asher G. (2002). Transdermal fentanyl for the management of acute pancreatitis pain. Appl. Nurs. Res..

[48] Chen Z., Jiang K., Liu F., Zhu P., Cai F., He Y., Jin T., Lin Z., Li Q., Hu C. (2022). Safety and efficacy of intravenous hydromorphone patient-controlled analgesia versus intramuscular pethidine in acute pancreatitis: An open-label, randomized controlled trial. Front. Pharmacol..

[49] Ona X.B., Comas D.R., Urrútia G. (2013). Opioids for acute pancreatitis pain. Cochrane Database Syst. Rev..

[50] Cai W., Liu F., Wen Y., Han C., Prasad M., Xia Q., Singh V.K., Sutton R., Huang W. (2021). Pain Management in Acute Pancreatitis: A Systematic Review and Meta-Analysis of Randomised Controlled Trials. Front. Med..

[51] Almulhim M., Almulihi Q., Almumtin H., Alghanim M., AlAbdulbaqi D., Almulihi F. (2023). The Efficacy and Safety of Using Opioids in Acute Pancreatitis: An Update on Systematic Review and Meta-Analysis. Med. Arch..

[52] Meng W., Yuan J., Zhang C., Bai Z., Zhou W., Yan J., Li X. (2013). Parenteral analgesics for pain relief in acute pancreatitis: A. systematic review. Pancreatology.

[53] Manrai M., Dawra S., Singh A.K., Jha D.K., Kochhar R. (2023). Controversies in the management of acute pancreatitis: An update. World J. Clin. Cases.

[54] Thavanesan N., White S., Lee S., Ratnayake B., Oppong K.W., Nayar M.K., Sharp L., Drewes A.M., Capurso G., De-Madaria E. (2022). Analgesia in the Initial Management of Acute Pancreatitis: A Systematic Review and Meta-Analysis of Randomised Controlled Trials. World J. Surg..

[55] Stein C. (2016). Opioid receptors. Annu. Rev. Med..

[56] Drewes A.M. (2016). Definition, diagnosis and treatment strategies for opioid-induced bowel dysfunction—Recommendations of the Nordic Working Group. Scand. J. Pain.

[57] Brock C., Olesen S.S., Olesen A.E., Frøkjaer J.B., Andresen T., Drewes A.M. (2012). Opioid-induced bowel dysfunction: Pathophysiology; management. Drugs.

[58] Wu S.D., Zhang Z.H., Jin J.Z., Kong J., Wang W., Zhang Q., Li D.Y., Wang M.F. (2004). Effects of narcotic analgesic drugs on human Oddi’s sphincter motility. World J. Gastroenterol..

[59] Meng J., Yu H., Ma J., Wang J., Banerjee S., Charboneau R., Barke R.A., Roy S. (2013). Morphine Induces Bacterial Translocation in Mice by Compromising Intestinal Barrier Function in a TLR-Dependent Manner. PLoS ONE.

[60] Barlass U., Dutta R., Cheema H., George J., Sareen A., Dixit A., Yuan Z., Giri B., Meng J., Banerjee S. (2018). Morphine worsens the severity and prevents pancreatic regeneration in mouse models of acute pancreatitis. Gut.

[61] Bálint E.R., Fűr G., Kui B., Balla Z., Kormányos E.S., Orján E.M., Tóth B., Horváth G., Szűcs E., Benyhe S. (2022). Fentanyl but Not Morphine or Buprenorphine Improves the Severity of Necrotizing Acute Pancreatitis in Rats. Int. J. Mol. Sci..

[62] Meerveld B.G.-V., Gardner C.J., Little P.J., Hicks G.A., Dehaven-Hudkins D.L. (2004). Preclinical studies of opioids and opioid antagonists on gastrointestinal function. Neurogastroenterol. Motil..

[63] Ashok A., Faghih M., Azadi J.R., Parsa N., Fan C., Bhullar F., Gonzalez F.G., Jalaly N.Y., Boortalary T., Khashab M.A. (2022). Morphologic Severity of Acute Pancreatitis on Imaging Is Independently Associated with Opioid Dose Requirements in Hospitalized Patients. Dig. Dis. Sci..

[64] Wu L.M., Pendharkar S.A., Asrani V.M., Windsor J.A., Petrov M.S. (2017). Effect of Intravenous Fluids and Analgesia on Dysmotility in Patients with Acute Pancreatitis: A Prospective Cohort Study. Pancreas.

[65] Elias A., Korytny A., Klein A., Khoury Y., Ben Hur D., Braun E., Azzam Z.S., Ghersin I. (2022). The Association Between Opioid Use and Opioid Type and the Clinical Course and Outcomes of Acute Pancreatitis. Pancreas.

[66] Knoph C.S., Joseph N., Lucocq J., Olesen S.S., Huang W., Dhar J., Samanta J., Talukdar R., Capurso G., Preatoni P. (2025). No definite associations between opioid doses and severity of acute pancreatitis—Results from a multicentre international prospective study. Pancreatology.

[67] Knoph C.S., Cook M.E., Novovic S., Hansen M.B., Mortensen M.B., Nielsen L.B.J., Høgsberg I.M., Salomon C., Neergaard C.E.L., Aajwad A.J. (2024). No Effect of Methylnaltrexone on Acute Pancreatitis Severity: A Multicenter Randomized Controlled Trial. Am. J. Gastroenterol..

[68] Knoph C.S., Hesthaven A.S., Cook M.E., Novovic S., Hansen M.B., Mortensen M.B., Nielsen L.B.J., Høgsberg I.M., Salomon C., Thorlacius-Ussing O. (2025). Gastrointestinal Transit Time Assessed Using a CT-Based Radiopaque Marker Method in Patients with Acute Pancreatitis During Methylnaltrexone Treatment. Neurogastroenterol. Motil..

[69] McGuire S.P., Anderson M.P., Maatman T.K., Roch A.M., Butler J.R., Ceppa E.P., House M.G., Nakeeb A., Nguyen T.K., Schmidt C.M. (2023). Opioid analgesia in necrotizing pancreatitis: Incidence and timing of a hidden crisis. Am. J. Surg..

[70] Yang A.L., Jin D.X., Srivoleti P., Banks P.A., McNabb-Baltar J. (2019). Are Opioid-Naive Patients with Acute Pancreatitis Given Opioid Prescriptions at Discharge?. Pancreas.

[71] Ahmed A., Yakah W., Freedman S.D., Kothari D.J., Sheth S.G. (2020). Evaluation of Opioid Use in Acute Pancreatitis in Absence of Chronic Pancreatitis: Absence of Opioid Dependence an Important Feature. Am. J. Med..

[72] Jabaudon M., Chabanne R., Sossou A., Bertrand P.M., Kauffmann S., Chartier C., Guérin R., Imhoff E., Zanre L., Brénas F. (2015). Epidural analgesia in the intensive care unit: An observational series of 121 patients. Anaesth. Crit. Care Pain Med..

[73] Jabaudon M., Belhadj-Tahar N., Rimmelé T., Joannes-Boyau O., Bulyez S., Lefrant J.Y., Malledant Y., Leone M., Abback P.S., Tamion F. (2018). Thoracic epidural analgesia and mortality in acute pancreatitis: A multicenter propensity analysis. Crit. Care Med..

[74] Wang Q., Fu B., Su D., Fu X. (2022). Impact of early thoracic epidural analgesia in patients with severe acute pancreatitis. Eur. J. Clin. Investig..

[75] Sasabuchi Y., Yasunaga H., Matsui H., Lefor A.K., Fushimi K., Sanui M. (2017). Epidural analgesia is infrequently used in patients with acute pancreatitis: A retrospective cohort study. Acta Gastroenterol. Belg..

[76] Sadowski S.M., Andres A., Morel P., Schiffer E., Frossard J.L., Platon A., Poletti P.A., Bühler L. (2015). Epidural anesthesia improves pancreatic perfusion and decreases the severity of acute pancreatitis. World J. Gastroenterol..

[77] Jabaudon M., Genevrier A., Jaber S., Windisch O., Bulyez S., Laterre P.-F., Escudier E., Sossou A., Guerci P., Bertrand P.-M. (2023). Thoracic epidural analgesia in intensive care unit patients with acute pancreatitis: The EPIPAN multicenter randomized controlled trial. Crit. Care.

[78] Tyagi A., Gupta Y.R., Das S., Rai G., Gupta A. (2019). Effect of segmental thoracic epidural block on pancreatitis induced organ dysfunction: A preliminary study. Indian J. Crit. Care Med..

[79] Cammarano C.A., Sandhu N.S., Villaluz J.E. (2021). Localizing the pain: Continuous paravertebral nerve blockade in a patient with acute pancreatitis. Am. J. Case Rep..

[80] Mantuani D., Luftig P.A.J., Herring A., Mian M., Nagdev A. (2020). Successful emergency pain control for acute pancreatitis with ultrasound guided erector spinae plane blocks. Am. J. Emerg. Med..

[81] Elkoundi A., Eloukkal Z., Bensghir M., Belyamani L., Lalaoui S.J. (2019). Erector Spinae Plane Block for Hyperalgesic Acute Pancreatitis. Pain Med..

[82] Martínez S.G., Gómez Facundo H., Deiros García C., Pueyo Periz E.M., Ribas Montoliu R., Coronado Llanos D., Masdeu Castellvi J., Martin-Baranera M. (2021). Transversus abdominis plane block in acute pancreatitis pain management. Gastroenterol. Hepatol..

[83] Tintara S., Shah I., Yakah W., Kowalczyk J.J., Sorrento C., Kandasamy C., Ahmed A., Freedman S.D., Kothari D.J., Sheth S.G. (2023). Comparison of Opioid-Based Patient-Controlled Analgesia with Physician-Directed Analgesia in Acute Pancreatitis: A Retrospective Cohort Study. Dig. Dis. Sci..

[84] Olesen S.S., Bouwense S.A.W., Wildersmith O.H.G., Van Goor H., Drewes A.M. (2011). Pregabalin reduces pain in patients with chronic pancreatitis in a randomized, controlled trial. Gastroenterology.

[85] Drewes A.M., Kempeneers M.A., Andersen D.K., Arendt-Nielsen L., Besselink M.G., Boermeester M.A., Bouwense S., Bruno M., Freeman M., Gress T.M. (2019). Controversies on the endoscopic and surgical management of pain in patients with chronic pancreatitis: Pros and cons!. Gut.

[86] Smiley M.M., Lu Y., Vera-Portocarrero L.P., Zidan A., Westlund K.N. (2004). Intrathecal Gabapentin Enhances the Analgesic Effects of Subtherapeutic Dose Morphine in a Rat Experimental Pancreatitis Model. Anesthesiology.

[87] Lu Y., Vera-Portocarrero L.P., Westlund K.N. (2003). Intrathecal Coadministration of D-APV and Morphine Is Maximally Effective in a Rat Experimental Pancreatitis Model. Anesthesiology.

[88] Chen Q., Vera-Portocarrero L.P., Ossipov M.H., Vardanyan M., Lai J., Porreca F. (2010). Attenuation of Persistent Experimental Pancreatitis Pain by a Bradykinin B2 Receptor Antagonist. Pancreas.

[89] Abed A., Minaiyan M., Safaei A., Taheri D. (2013). Effect of Diazepam on Severity of Acute Pancreatitis: Possible Involvement of Peripheral Benzodiazepine Receptors. ISRN Gastroenterol..

[90] Jang D.K., Lee J.K., Jung C.Y., Kim K.H., Kang H.R., Lee Y.S., Yoon J.H., Joo K.R., Chae M.K., Baek Y.H. (2023). Electroacupuncture for abdominal pain relief in patients with acute pancreatitis: A three-arm randomized controlled trial. J. Integr. Med..

[91] Zhu F., Yin S., Zhu X., Che D., Li Z., Zhong Y., Yan H., Gan D., Yang L., Wu X. (2021). Acupuncture for Relieving Abdominal Pain and Distension in Acute Pancreatitis: A Systematic Review and Meta-Analysis. Front. Psychiatry.

[92] Deng L., Chen Z., Jin T., Cai F., He Y., Shen Y., Zhang S., Guo J., Yang X., Yang L. (2025). Traditional Chinese medicine Chaiqinchengqi decoction for patients with acute pancreatitis: A randomized clinical trial. Phytomedicine.

[93] Joseph N., Kuhlmann L., Lucocq J., Aulenkamp J., Knoph C.S., Olesen S.S., Pogatzki-Zahn E.M., Windsor J.A., Drewes A.M., Pandanaboyana S. (2025). A Systematic Review of Pain Assessment Domains in Acute Pancreatitis Randomised Controlled Trials. Pancreas.

[94] Kuhlmann L., Pogatzki-Zahn E.M., Joseph N., Lucocq J., Aulenkamp J., Knoph C.S., Olesen S.S., Windsor J.A., Drewes A.M., Pandanaboyana S. (2025). Development and Validation of the Comprehensive Acute Pancreatitis Pain Core Outcome Set (CAPPOS): Study Protocol. Pancreas.

[95] Sweeney S., Crowe S., Watson W., Rasmussen B., Wynter K., Holton S. (2020). End PJ Paralysis: An initiative to reduce patient’s functional decline. Aust. Nurs. Midwifery J..

[96] Capurso G., Ponz de Leon Pisani R., Lauri G., Archibugi L., Hegyi P., Papachristou G.I., Pandanaboyana S., Maisonneuve P., Arcidiacono P.G., De-Madaria E. (2023). Clinical usefulness of scoring systems to predict severe acute pancreatitis: A systematic review and meta-analysis with pre and post-test probability assessment. United Eur. Gastroenterol. J..

